# Impact of structured medication reviews on prescribing in English primary care: a retrospective observational cohort study

**DOI:** 10.3399/BJGP.2025.0617

**Published:** 2026-06-16

**Authors:** James P Sheppard, Paul A Bateman, Cynthia Wright-Drakesmith, Christopher Elles Clark, Rebecca K Barnes, Andrew Paul Clegg, Gary A Ford, Seema Gadhia, William Hinton, FD Richard Hobbs, Sundus Jawad, Kamlesh Khunti, Gregory YH Lip, Simon de Lusignan, Jonathan Mant, Deborah McCahon, Bernardo Meza-Torres, Rupert A Payne, Rafael Perera-Salazar, Claire Reidy, Anna Seeley, Samuel Seidu, Katherine Tucker, Rik van der Veen, Marney Williams, Richard J McManus

**Affiliations:** 1 Nuffield Department of Primary Care Health Sciences, University of Oxford, Oxford, UK; 2 Exeter Collaboration for Academic Primary Care, University of Exeter, Exeter, UK; 3 Academic Unit for Ageing & Stroke Research, University of Leeds, Leeds, UK; 4 Bradford Institute for Health Research, Bradford Teaching Hospitals NHS Foundation Trust, Bradford, UK; 5 Health Innovation Oxford and Thames Valley, Oxford, UK; 6 Radcliffe Department of Medicine, University of Oxford, Oxford, UK; 7 Oxford Institute of Digital Health, University of Oxford, Oxford, UK; 8 NHS Frimley Integrated Care Board, Windsor, UK; 9 Diabetes Research Centre, University of Leicester, Leicester, UK; 10 Institute of Life Course and Medical Sciences, University of Liverpool, Liverpool, UK; 11 Clinical Department of Cardiology, Lipidology and Internal Medicine with Cardiac Intensive Care Unit, Medical University of Białystok, Białystok, Poland; 12 Primary Care Unit, Department of Public Health and Primary Care, University of Cambridge, Cambridge, UK; 13 Centre for Academic Primary Care, Population Health Sciences, University of Bristol, Bristol, UK; 14 patient and public advisor, London, UK; 15 Brighton and Sussex Medical School, University of Brighton and University of Sussex, Brighton, UK

**Keywords:** deprescribing, electronic health records, inappropriate prescribing, polypharmacy, primary health care, structured medication review

## Abstract

**Background:**

Structured medication reviews (SMRs) were introduced in 2020 to address polypharmacy in patients most at risk of medicines-related harm.

**Aim:**

To evaluate the impact of SMRs on prescribing in primary care.

**Design and setting:**

Retrospective observational cohort study of electronic health records from patients aged ≥65 years, prescribed ≥1 medications, and fulfilling the specific eligibility criteria for an SMR, registered at practices contributing data to the Oxford Clinical Informatics Digital Hub, between 1 April 2020 and 30 September 2022.

**Method:**

The association between SMRs and prescription changes was examined by matching individuals who received an SMR to individuals who did not receive an SMR, according to age, sex, and primary care practice, using cumulative density sampling. Analyses were undertaken using adjusted logistic regression.

**Results:**

Of 635 698 eligible patients, 82 285 (12.9%, 95% confidence interval [CI] = 12.9 to 13.0) received ≥1 SMR during the study observation period. In those prescribed potentially inappropriate drug combinations prior to an SMR, between 12.5% and 40.0% were corrected up to 3 months later. In matched analyses, SMRs were most strongly associated with an increase in new prescriptions of angiotensin-converting enzyme inhibitors (adjusted odds ratio [aOR] 1.56, 95% CI = 1.35 to 1.81), statins (aOR 1.78, 95% CI = 1.57 to 2.02), and antidepressants (aOR 1.45, 95% CI = 1.28 to 1.63). SMRs were also most strongly associated with stopping these drug classes in those previously prescribed treatment.

**Conclusion:**

SMRs were associated with starting new medications and stopping existing prescriptions compared with usual care. Further work is needed to understand if these changes improved patient outcomes.

**How this fits in**

Structured medication reviews (SMRs) are a National Institute for Health and Care Excellence approved clinical intervention to address complex or problematic polypharmacy and were introduced widely in the NHS in 2020. This study found that one in eight eligible patients received an SMR during the first 2 years of the programme’s rollout in England. SMRs were associated with an increased likelihood of starting medication in those not previously prescribed treatment and an increased likelihood of stopping medications in those with existing prescriptions. This analysis was limited by the data available within primary care electronic health records and so it is unclear if the observed changes in prescribing resulted in improvements in patient outcomes.

## Introduction

Prescribing medicines is the most common intervention in the NHS, and most prescribing takes place in primary care.^1,2^ However, inappropriate polypharmacy (where medicines are no longer appropriate)^3^ can expose the most vulnerable patients to decreased quality of life^4–6^ and hospital admissions with adverse drug events.^7–9^ It is estimated that nearly £400 million is spent each year on admissions to hospital caused by harm from potentially inappropriate medication prescriptions.^10^

Over the past 20 years, initiatives have sought to reduce polypharmacy-related harm in UK primary care, and the National Institute for Health and Care Excellence has recommended structured medication reviews (SMRs) as a clinical intervention to address complex or problematic polypharmacy.^11^ Historically, medication reviews were undertaken by GPs, but SMRs were designed to be undertaken by clinical pharmacists embedded in primary care, reviewing medications prescribed to patients most at risk of medicines-related harm.^11,12^ This was based on evidence from studies such as the PINCER trial,^13^ which showed benefits from a pharmacist-led approach to reduce hazardous prescribing in primary care. SMRs were introduced via primary care networks (PCNs; groups of neighbouring GP practices in England, typically serving 30 000–50 000 people, formed to work together to deliver more proactive, integrated care) in 2020,^12^ with participating GPs receiving funding from their local PCN to employ practice-based pharmacists to deliver SMRs, and further financial incentives based on the number of SMRs undertaken and the achievement of medicines optimisation within these reviews. Each year, the specification for SMRs was slightly updated, with certain populations and optimisation targets prioritised (for example, people in nursing homes and those with severe frailty, multimorbidity, and/or polypharmacy were targeted from October 2021).

Clinical trials focused on chronic diseases have shown that SMRs can improve cardiovascular risk management. However, these studies vary widely in intervention types and measured outcomes.^14^ Furthermore, there is limited evidence that medication reviews reduce adverse drug events.^15,16^ Evaluation studies of pharmacist-led medication reviews undertaken in routine clinical practice have shown mixed results in terms of prescribing and patient-centred care.^17–19^ One possible reason for this is that, historically, primary care practices have prioritised being time-efficient rather than being comprehensive, so that most medication reviews were carried out with little or no patient involvement, and medicines were rarely stopped or reduced.^20^ In an early qualitative evaluation undertaken during the first year following the introduction of SMRs, it was suggested that the implementation of the service had been suboptimal, failing to match the aspiration for patients that was presented in the original policy.^21^

To date, to the authors’ knowledge, quantitative evaluations of the service have been limited to descriptive analyses of who received an SMR during the COVID-19 pandemic.^22^ The impact of the SMR programme on prescribing in primary care remains unknown. The present study therefore aimed to evaluate the impact of SMRs on prescribing in primary care by determining the proportion of eligible patients that received an SMR in the first 2 years and whether SMRs were associated with changes in prescribing and primary care contacts during this period.

## Method

Detailed extended methods are provided in Supplementary Information S1.

### Setting

This was a retrospective observational cohort study using routine data from primary care electronic health records (EHRs) from GP practices in England collected via the Oxford Clinical Informatics Digital Hub (ORCHID).^23,24^ This database contains all coded data regarding medical history, prescriptions, and test results from over 1800 general practices across England. ORCHID includes data from multiple clinical software systems including EMIS, SystmOne, and Vision. The database represents over a quarter of English general practices and 30% of the English population, and has been shown to be representative of patients in England in terms of age, sex, ethnicity, socioeconomic status, and geographical spread.^23^

### Population

Eligible patients were aged ≥65 years, prescribed ≥1 repeat medications, and who fulfilled at least one of the eligibility criteria for an SMR, as defined in the PCN contract for SMRs.^25^ Individuals entered the cohort on 1 January 2020 and SMRs that occurred between 1 April 2020 and 30 September 2022 were included. Data from the 3 months before the SMR observation period (January to March 2020) were used to define treatment prescriptions. Medication changes (new prescriptions and stoppages of existing prescriptions) up to 3 months after an SMR were included up until 31 December 2022.

### Outcomes

The primary outcome of this study was the proportion of eligible patients receiving an SMR during the SMR observation period. Secondary outcomes included changes in medication prescriptions (new prescriptions and stoppages of existing prescriptions) following an SMR. Further analyses focused on changes in medications identified in the PCN contract as being potentially inappropriate (see Supplementary Table S1 for details). Finally, changes in any primary care contacts between healthcare professionals and patients before and after an SMR were examined. For this analysis, healthcare professionals were defined as GPs, pharmacists, healthcare assistants (HCAs), or nurses.

### Exposures

The exposure of interest was an SMR during the observation period 1 April 2020 to 30 September 2022, defined using the Systematized Nomenclature of Medicine clinical term 1239511000000100.

### Covariates

All analyses examining the association between SMRs and outcomes were adjusted for covariates defined according to data available at any timepoint prior to the observation period (that is, before April 2020) and included body mass index, ethnicity, Index of Multiple Deprivation (IMD), smoking status, care home residence, baseline cholesterol, electronic Frailty Index score,^26^ and number of multiple long-term conditions. The latter were defined based on the list of 37 conditions included in the Cambridge Multimorbidity Score.^27,28^ Age (defined at the time of the index date) and sex were not included in the modelling as these were used as matching variables for the cohort.

### Statistical analysis

Descriptive analyses were used to determine the proportion of patients potentially eligible to receive an SMR during the study observational period. In those who received an SMR and had continuous follow-up (that is, those who survived and remained within ORCHID-registered practices so that medication prescription could be measured), changes in medication prescription (stopped or started) within 3 months (91 days) of an SMR were examined according to individual drug class. Furthermore, potentially inappropriate medication prescriptions were examined by identifying individuals with specific conditions or concurrent drug prescriptions that would put them at risk of adverse drug events, as detailed in the PCN contract for SMRs (see Supplementary Table S1 for details).^29^ The proportion of these potential medication errors that were corrected within 3 months of an SMR was estimated using descriptive statistics.

To examine the associations between SMRs and prescription changes and primary care contacts, a cohort of patients who received an SMR were matched to control patients who did not receive an SMR using cumulative density sampling. Individuals were matched 1:1 according to baseline age (5-year groups), sex, and primary care practice. Each control patient was given an index date that was the date their matched case patient received an SMR. Analyses of the association between SMRs and outcomes were undertaken using logistic regression models, which were adjusted for baseline covariates (listed above), with missing data for IMD dealt with using multiple imputation (10 imputations). All analyses were undertaken using Stata (version 18.0).

## Results

A total of 635 698 patients from 783 practices across England were identified as being eligible for an SMR. Of these, 82 285 patients (12.9%, 95% confidence interval [CI] = 12.9 to 13.0) received ≥1 SMR during the study observational period. More patients received an SMR in the second year of the observation period than the first year (see Supplementary Figure S1). Of the 82 285 patients who received an SMR, they were on average aged 77 years, 59.5% (*n* = 48 997) were female, with 3.3% (*n* = 2745) of Asian ethnicity and 1.6% (*n* = 1326) of Black ethnicity (Table 1). Compared with those patients who did not receive an SMR, there were higher proportions of residents in nursing homes, patients with moderate-to-severe frailty, with hyper-polypharmacy (≥10 prescriptions), and with complex multiple long-term conditions (≥4 conditions present) in those who did receive one (Table 1 and Supplementary Tables S2 and S3). There were no observed differences in the proportion of patients receiving an SMR on the basis of age, sex, ethnicity, or social deprivation.

**Table 1.** Baseline characteristics of those receiving and not receiving an SMR (*N* = 635 698)

Characteristic	Received an SMR, *n* (%)^a^	Did not receive an SMR, *n* (%)^a^
**Total cohort population**	82 285 (12.9)	553 413 (87.1)
**Age at index date, years, median (IQR)**	77 (72–84)	76 (71–83)
**Sex**, **female**	48 997 (59.5)	316 779 (57.2)
**Ethnicity**		
Asian	2745 (3.3)	15 952 (2.9)
Black	1326 (1.6)	6906 (1.2)
Mixed	281 (0.3)	1887 (0.3)
Other	337 (0.4)	2170 (0.4)
White	73 027 (88.7)	478 213 (86.4)
Unknown	4569 (5.6)	48 285 (8.7)
**BMI, mean (SD)**	28.5 (6.2)	28.0 (6.0)
**Smoking status**		
Active smoker	8443 (10.3)	59 412 (10.7)
Ex-smoker	34 224 (41.6)	229 455 (41.5)
Non-smoker	38 082 (46.3)	254 902 (46.1)
Missing	1536 (1.9)	9644 (1.7)
**IMD quintile**		
1 (most deprived)	12 580 (15.3)	76 084 (13.7)
2	14 675 (17.8)	92 435 (16.7)
3	16 606 (20.2)	108 167 (19.5)
4	16 278 (19.8)	117 894 (21.3)
5 (least deprived)	15 579 (18.9)	118 462 (21.4)
Missing	6567 (8.0)	40 371 (7.3)
**Location**		
Living in a rural area	18 590 (22.6)	125 805 (22.7)
Living in an urban area	59 331 (72.1)	401 094 (72.5)
Missing	4364 (5.3)	26 514 (4.8)
**Blood pressure**		
Systolic, mmHg, mean (SD)	132.7 (17.0)	133.3 (17.1)
Missing	41 478 (50.4)	332 018 (60.0)
Diastolic, mmHg, mean (SD)	74.2 (10.0)	74.5 (10.1)
Missing	44 039 (53.5)	346 540 (62.6)
**Cholesterol**		
Total cholesterol, mM, mean (SD)	4.49 (1.18)	4.61 (1.29)
Missing	2204 (2.7)	16 807 (3.0)
HDL cholesterol, mM, mean (SD)	1.45 (0.45)	1.48 (0.63)
Missing	2970 (3.6)	22 734 (4.1)
**Residing in a care home**	8209 (10.0)	25 884 (4.7)
**Frailty**		
Fit	5991 (7.3)	79 774 (14.4)
Mild	24 450 (29.7)	202 346 (36.6)
Moderate	27 770 (33.7)	160 602 (29.0)
Severe	24 074 (29.3)	110 691 (20.0)
**eFI score**		
≤0.36 (not severe)	64 122 (77.9)	471 827 (85.3)
>0.36 (severe)	18 163 (22.1)	81 586 (14.7)
**Polypharmacy**		
0–4	3297 (4.0)	50 664 (9.2)
5–9	21 475 (26.1)	187 579 (33.9)
≥10	57 513 (69.9)	315 170 (57.0)
**Number of MLTC**		
0–1	2803 (3.4)	34 411 (6.2)
2–3	15 364 (18.7)	142 428 (25.7)
≥4	64 118 (77.9)	376 574 (68.0)

^a^Unless otherwise specified. BMI = body mass index. eFI = electronic Frailty Index. IMD = Index of Multiple Deprivation. IQR = interquartile range. MLTC = multiple long-term conditions. SD = standard deviation. SMR = structured medication review.

The majority of patients had no changes in their treatment following an SMR (Table 2). In those already prescribed treatment, the medications most commonly stopped were non-steroidal anti-inflammatory drugs (NSAIDs) (*n* = 3882/13 102; 29.6% stopped), benzodiazepines (*n* = 1130/5073; 22.3%), laxatives (*n* = 3427/17 205; 19.9%), Z-drugs (*n* = 637/3946; 16.1%), and opioids (*n* = 4230/27 364; 15.5%). In patients not previously taking a given drug therapy, proton pump inhibitors (PPIs) (*n* = 4075/35 948; 11.3% started), statins (*n* = 2662/34 389; 7.7%), opioids (*n* = 4197/54 921; 7.6%), and laxatives (*n* = 4194/65 080; 6.4%) were most likely to be started, although the proportion starting treatment was smaller (compared with the proportion stopping).

**Table 2.** Changes in medication prescription following an SMR (*N* = 82 285)

Medication prescription	Total on prescription before an SMR, *n*	Prescription stopped after SMR, *n* (%)	Total not on prescription before an SMR, *n*	Prescription started after SMR, *n* (%)
**Antihypertensives**				
ACE inhibitors	23 282	1188 (5.1)	59 003	1111 (1.9)
Alpha blockers	5108	379 (7.4)	77 177	472 (0.6)
Angiotensin II receptor blockers	14 376	619 (4.3)	67 909	733 (1.1)
Beta blockers	28 403	973 (3.4)	53 882	1325 (2.5)
Calcium channel blockers	25 304	1545 (6.1)	56 981	1747 (3.1)
Thiazide and thiazide-like diuretics	7407	774 (10.4)	74 878	584 (0.8)
**Other cardiovascular medications**				
Statins	47 896	1752 (3.7)	34 389	2662 (7.7)
Aspirin	16 958	1438 (8.5)	65 327	1094 (1.7)
Other antiplatelets	10 918	890 (8.2)	71 367	780 (1.1)
DOAC	16 822	600 (3.6)	65 463	1467 (2.2)
Vitamin K antagonists	3284	338 (10.3)	82 142	143 (0.2)
**Inhaled medication**				
Inhaled beta agonist	15 279	1226 (8.0)	67 006	1436 (2.1)
Inhaled corticosteroids	14 188	1491 (10.5)	68 097	1648 (2.4)
**Pain medications**				
NSAIDs	13 102	3882 (29.6)	69 183	3934 (5.7)
Opioids	27 364	4230 (15.5)	54 921	4197 (7.6)
**Other medications**				
Antidepressants	27 675	1828 (6.6)	54 610	1923 (3.5)
Benzodiazepines	5073	1130 (22.3)	77 212	1324 (1.7)
Donepezil	2639	95 (3.6)	79 646	159 (0.2)
Gabapentin	5856	598 (10.2)	76 429	519 (0.7)
Pregabalin	4335	380 (8.8)	77 950	458 (0.6)
Z-drugs	3946	637 (16.1)	78 339	681 (0.9)
Laxatives	17 205	3427 (19.9)	65 080	4194 (6.4)
PPI	46 337	2414 (5.2)	35 948	4075 (11.3)

ACE = angiotensin-converting enzyme. DOA = direct oral anticoagulant. NSAIDs = non-steroidal anti-inflammatory drugs. PPI = proton pump inhibitor. SMR = structured medication review.

With regard to potentially inappropriate prescriptions, between 12.5% and 40.0% were corrected, although the overall numbers of inappropriate prescriptions were small (Table 3). Medication combinations that increase the risk of gastrointestinal bleeds were most commonly corrected (between 22.9% and 40.0% of combinations corrected), whereas drugs that could exacerbate asthma (12.5% corrected) or lead to respiratory depression (14.9% corrected) were least commonly corrected.

**Table 3.** Changes in potentially inappropriate medication prescriptions following a structured medication review

Indicator	Condition	Drug or drug combination	Total with inappropriate medication combinations, *n*	Not corrected following SMR, ***n*** (%)	Corrected following SMR, *n* (%)
GIB01	GI bleed	NSAID, but not PPI	101	65 (64.4)	36 (35.6)
GIB02	GI bleed	NSAID and anticoagulant	190	114 (60.0)	76 (40.0)
GIB03	GI bleed	NSAID and anticoagulant, but not PPI	35	27 (77.1)	8 (22.9)
GIB04	GI bleed	Aspirin and antiplatelet, but not PPI	93	64 (68.8)	29 (31.2)
GIBCI	GI bleed	Unique patients from GIB01 to GIB04	324	201 (62.0)	123 (38.0)
PAIN01	Respiratory depression/confusion	Opioid and benzo/gabapentin/ pregabalin/Z-drug	72	57 (79.2)	15 (20.8)
PAIN02	Constipation	Opioid, but not laxative	1001	766 (76.5)	235 (23.5)
PAIN03	Respiratory depression/confusion	Opioid	202	172 (85.1)	30 (14.9)
FRAC01a	Fall	Z-drug	1218	1002 (82.3)	216 (17.7)
FRAC01b	Fracture	Z-drug	276	226 (81.9)	50 (18.1)
FRAC02a	Fall	Benzo	1741	1295 (74.4)	446 (25.6)
FRAC02b	Fracture	Benzo	456	331 (72.6)	125 (27.4)
FRAC03a	Fall	Benzo/Z-drug	191	119 (62.3)	72 (37.7)
FRAC03b	Fracture	Benzo/Z-drug	687	480 (69.9)	207 (30.1)
RESP01	Asthma	LABA, but not inhaled corticosteroid	112	98 (87.5)	14 (12.5)

Benzo = benzodiazepine. DOAC = direct oral anticoagulant. GI  = gastrointestinal. LABA  = inhaled long-acting beta agonist. NSAID = non-steroidal anti-inflammatory drug. PPI = proton pump inhibitor. SMR = structured medication review.

**Figure 1.**
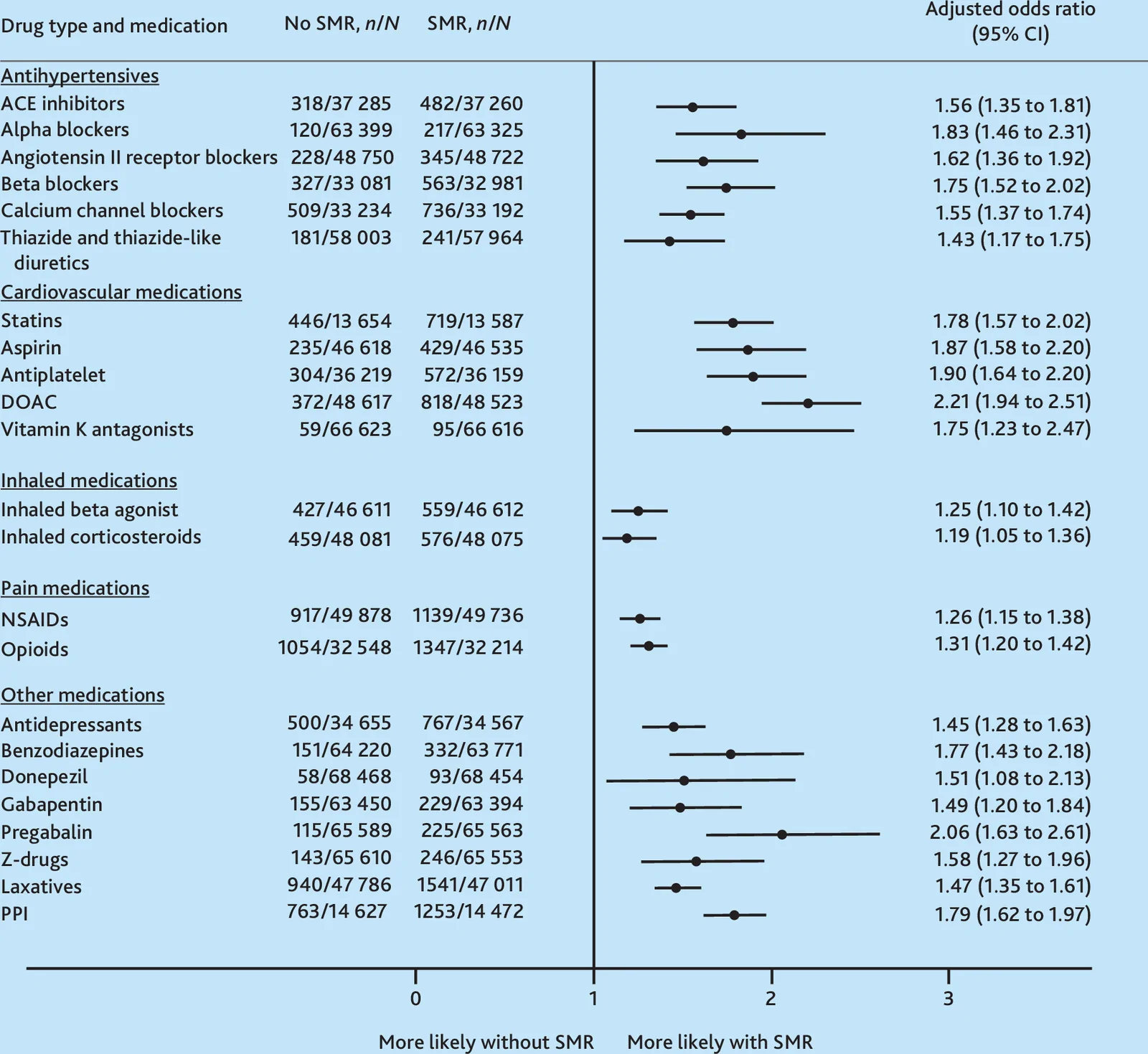
Association between structured medication reviews (SMRs) and starting medications for the first time. Models adjusted for body mass index, ethnicity, Index of Multiple Deprivation, smoking status, care home residence, baseline cholesterol, electronic Frailty Index score,^26^ and number of multiple long-term conditions.^27^ ACE = angiotensin-converting enzyme. DOAC = direct oral anticoagulant. NSAIDs = non-steroidal anti-inflammatory drugs. PPI = proton pump inhibitor.

**Figure 2.**
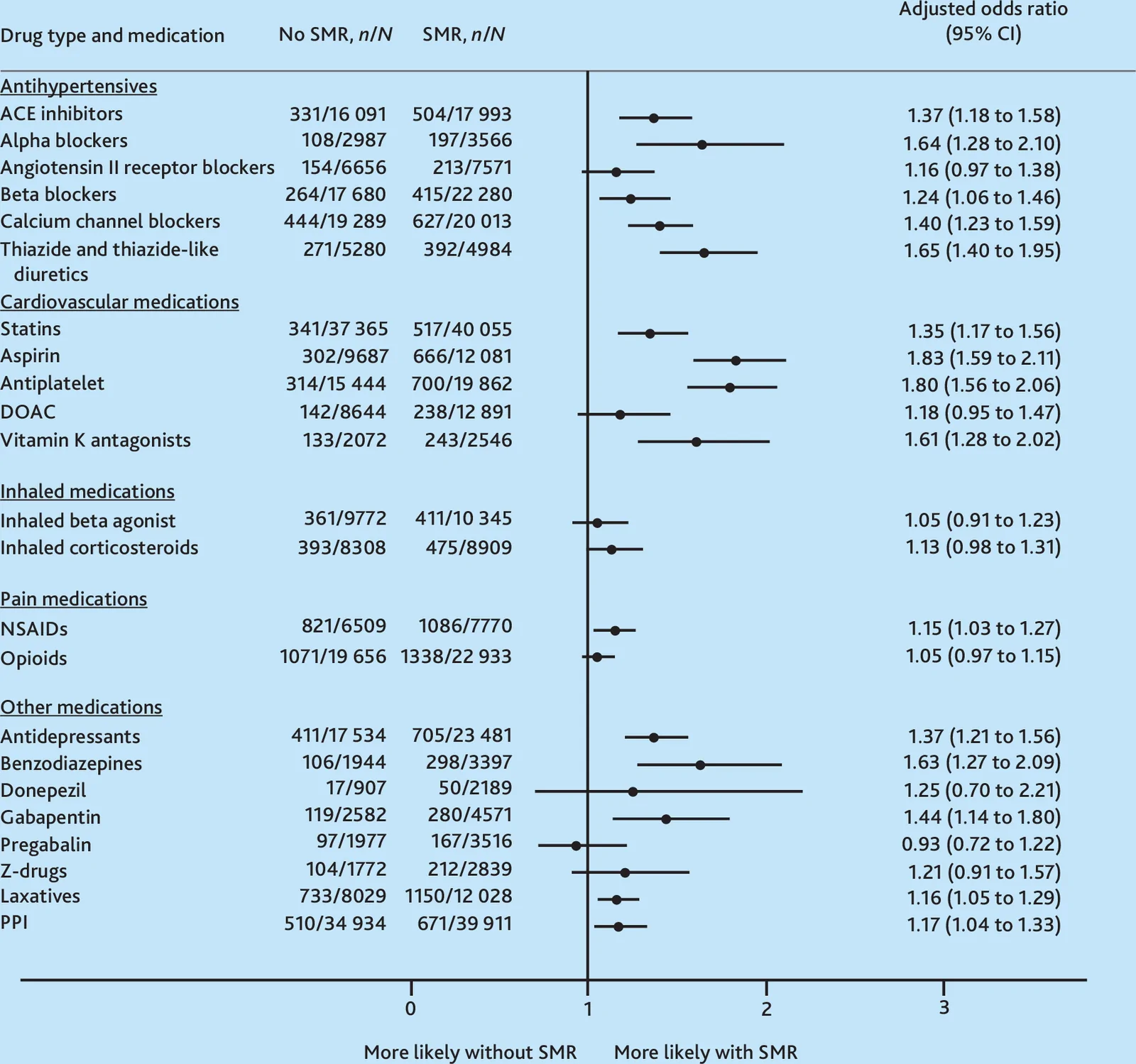
Association between structured medication reviews (SMRs) and stopping medications prescribed before the index date. Models adjusted for body mass index, ethnicity, Index of Multiple Deprivation, smoking status, care home residence, baseline cholesterol, electronic Frailty Index score,^26^ and number of multiple long-term conditions.^27^ ACE = angiotensin-converting enzyme. DOAC = direct oral anticoagulant. NSAIDs = non-steroidal anti-inflammatory drugs. PPI = proton pump inhibitor.

In 71 939 patients who received an SMR, matched to 71 939 patients who did not (see Supplementary Table S4), medication reviews were associated with a significant increase in starting new medications and stopping existing prescriptions across nearly all drug classes of interest. This included increased prescribing of antihypertensives, cardiovascular, inhaled, pain, psychotropic, and other medications in patients receiving an SMR (Figure 1). Furthermore, those already prescribed antihypertensives, cardiovascular, psychotropic, laxatives, and PPIs were more likely to have them stopped if they received an SMR (Figure 2). There was no evidence of an association between SMRs and stopping inhaled or pain medications, with the exception of NSAIDs.

Patients receiving an SMR had an average of four (interquartile range [IQR] 2–7) primary care contacts with clinical personnel in the 3 months before their review, and five (IQR 3–8) contacts in the 3 months after. In contrast, those not receiving an SMR had an average of three (IQR 1–5) contacts before and after the index date, meaning that SMRs were associated with a significant increase in primary care contacts of 0.14 (95% CI = 0.13 to 0.16; equivalent to 14 extra patient contacts for every 100 individuals receiving an SMR). Results were similar in analyses using different definitions to describe primary care contacts, including those where contacts with a code for an SMR were excluded (see Supplementary Table S5).

## Discussion

### Summary

In this analysis, one in eight eligible patients received an SMR in the first 2 years of the SMR service rollout in England. This reflected a time of significant change for the NHS as initial implementation coincided with the first winter of the COVID-19 pandemic alongside major changes in staffing including a large increase in the numbers of pharmacists employed in primary care.^30^ SMRs that were undertaken appeared appropriately targeted towards patients with greater frailty and multiple long-term conditions, without any clear demographic or socioeconomic bias.

As might be expected, many people continued with their prescribed medication following an SMR, but modest net changes in prescribing masked a significant increase in starting new medications and stopping existing prescriptions across nearly all drug classes of interest. In particular, SMRs were associated with an increased likelihood of stopping preventative medications, NSAIDs, and some psychotropic medications such as antidepressants, benzodiazepines, and gabapentin. However, for other psychotropic medications, such as pregabalin and Z-drugs, and pain medications, such as opioids, there was no assocation between SMRs and stopping these medications, reflecting clinical caution or patient resistance to discontinuing medications that are prescribed for persistent symptoms despite limited evidence for long-term benefit.

### Strengths and limitations

This was a large nationwide observational study including SMRs undertaken across England covering the first 2 years of the service. The completion and coding of SMRs was incentivised through the Investment and Impact Fund during the period of this study, meaning that the study’s estimates of SMR completion from the EHRs are likely to be accurate. The large sample size available meant that medication prescription changes before and after an SMR could be examined across a range of drug classes. Almost all medication prescriptions are electronic and automatically coded in English primary care health records and therefore these data accurately reflect prescribing practice. However, the present analyses were unable to take into account changes in dosing, likely to be an important aspect of an SMR, which tend to be recorded in free-text fields, which were not available for this study.

Analyses of inappropriate medication indicators included prescriptions of certain drug combinations, in the presence of medical conditions that would make prescription inappropriate. Many of these scenarios required information about whether an individual had recently attended hospital with an acute event (for example, a fall or gastrointestinal bleed). However, as linked hospital data were not available, and information about acute events occurring in secondary care is not well coded in GP records,^31,32^ this aspect of the analysis should be interpreted with caution.

The before and after study design defined medication changes in the 3 months before and 3 months after an SMR. This was chosen as representing the longest time period that repeat prescriptions of medications for long-term conditions are likely to be prescribed.^33^ However, medication prescribing patterns are rarely uniform and therefore defining the exposures using shorter or longer time frames may have resulted in slightly different findings.

### Comparison with existing literature

Few studies have sought to determine the impact of SMRs on prescribing practices in primary care. One previous study using routine EHR data from the OpenSafely database observed a lower percentage of patients (3.6%) receiving SMRs;^22^ however, that study had a broader focus on all types of medication review, and the denominator population was not limited to patients eligible for an SMR.

Approximately one-third of inappropriate medication combinations associated with gastrointestinal bleeding (NSAIDs, anticoagulants, aspirin, and antiplatelets but not PPIs) were corrected following an SMR in the present study. Similar findings were observed in a recent study examining the scale-up and rollout of the PINCER intervention, a pharmacist-led approach to reduce hazardous prescribing in primary care.^13^ Across 343 general practices implementing the intervention in routine clinical care, decreases in inappropriate prescribing were observed for all indicators, including a 24% reduction in medication combinations associated with gastrointestinal bleeding.^34^

Evidence from randomised controlled trials suggests that medication reviews result in changes in prescribing and some surrogate clinical endpoints such as blood pressure and cholesterol;^14^ however, there is no evidence that they reduce mortality, hospital admissions, healthcare use, and number of patients falling, or improve physical and cognitive functioning and quality of life.^15,16,35^ The present analysis shows that these effects on prescribing could be realised in routine clinical practice as well; however, the effect on clinical and cost-effectiveness outcomes remains unknown.

### Implications for research and practice

The present study found evidence that SMRs are associated with significant increases in starting new medications and stopping existing prescriptions in vulnerable patients with frailty, multiple long-term conditions, and polypharmacy. There was evidence that some inappropriate medication combinations, such as those related to an increased risk of gastrointestinal bleeding, were corrected following an SMR, although the majority were still prescribed in the 3 months after an SMR. Addressing such inappropriate prescriptions could have significant consequences for the healthcare system, where recent analyses suggest that inappropriate prescribing of NSAIDs alone (without PPIs) could cost the NHS £2.46 million per year, with a loss of nearly 2000 quality-adjusted life years.^36^

SMRs were associated with a small increase in primary care contacts, defined as face-to-face or telephone/video appointments with GPs, pharmacists, HCAs, or nurses. Whether this is to be expected is unclear; however, the PCN contract specification for SMRs^29^ does outline expectations for follow-up of SMRs, which could explain the observed increase in primary care contacts.

Despite first being rolled out during the COVID-19 pandemic, one in eight eligible patients received an SMR, with evidence of targeting patients with greater frailty, more polypharmacy, and larger numbers of long-term conditions, with no evidence of demographic or socioeconomic bias. This suggests that SMRs were targeted as intended at those most likely to be at risk of inappropriate polypharmacy and adverse drug events.^37^

Although this study showed that SMRs were associated with changes in prescribing and deprescribing following an SMR, the effect on clinically relevant patient outcomes remains unknown. Future studies should utilise linked data from both primary and secondary care to examine the association between SMRs and clinical outcomes, such as hospital admission because of adverse drug events. Such analyses will require careful adjustment for confounding by indication and classification of outcomes that may be captured with varying accuracy. Furthermore, it remains unclear whether the investment in practice-based pharmacists to deliver SMRs is a cost-effective approach, and health economic modelling of this service would provide valuable information for policymakers in the future.

In conclusion, this study found that one in eight eligible patients received an SMR within the first 2 years of the service rollout. SMRs were associated with a significant increase in starting new medications and stopping existing prescriptions across nearly all drug classes of interest. However, further work is needed to understand whether these changes in prescribing improved patient outcomes in the longer term.

## Data

Requests for data sharing should be made directly to the Primary Care Hosted Research Datasets Independent Scientific Committee, based at the University of Oxford. Code lists used to define variables included in the dataset are available at: https://github.com/ndpchs-cprd/PD-0002-2022-OSCAR.
